# Peak frequency analysis to identify critical isthmus distribution for adenosine triphosphate–sensitive atrial tachycardia

**DOI:** 10.1016/j.hroo.2026.04.006

**Published:** 2026-04-15

**Authors:** Satoshi Oka, Yuichiro Miyazaki, Koji Miyamoto, Akinori Wakamiya, Nobuhiko Ueda, Kenzaburo Nakajima, Kosuke Nakasuka, Tsukasa Kamakura, Mitsuru Wada, Yuko Inoue, Kengo Kusano

**Affiliations:** Department of Cardiovascular Medicine, National Cerebral and Cardiovascular Center, Suita, Japan

**Keywords:** ATP-sensitive atrial tachycardia, Catheter ablation, Microreentrant circuit, Peak frequency, Critical isthmus identification

**Figure:**
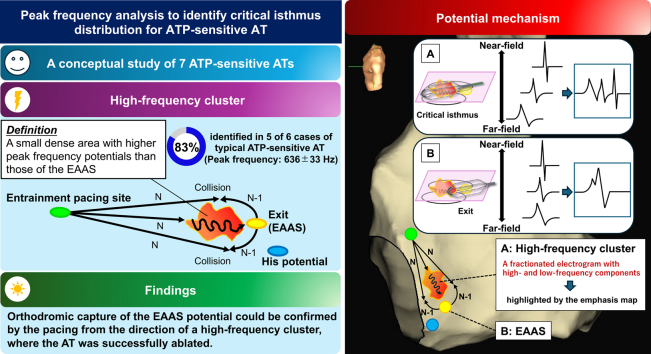

**Key Findings**

▪ Among 6 typical adenosine triphosphate–sensitive atrial tachycardias (ATs), a high-frequency cluster adjacent to the earliest atrial activation site (EAAS) was identified in 5 cases.
▪ Orthodromic capture of the EAAS potential was confirmed by pacing from the direction of a high-frequency cluster, where AT was successfully ablated.
▪ The absence of a high-frequency cluster or a relatively low peak frequency at the EAAS in the right atrium may warrant additional mapping of the left atrium or the noncoronary cusp.

## Introduction

Adenosine triphosphate (ATP)–sensitive atrial tachycardia (AT) typically arises from the Koch’s triangle. The underlying mechanism has been elucidated as a reentry,^1^ involving calcium channel–dependent tissues within the circuit. The critical isthmus distribution can be identified using entrainment pacing delivered from multiple sites of the right atrium (RA). If orthodromic capture of the earliest atrial activation site (EAAS) is confirmed, radiofrequency (RF) application from the direction suppresses the AT, offering sufficient distance from the cardiac conduction system. However, we cannot evaluate the critical isthmus direction if the entrainment pacing repeatedly terminates the AT. Furthermore, visualizing microreentrant circuits remains challenging, despite the high-density 3-dimensional (3D) mapping system.

Omnipolar technology (OT) captures true electrograms independent of catheter orientation relative to the wavefront using an Advisor HD Grid™ catheter and the EnSite™ X 3D mapping system (Abbott, Abbott Park, IL). Furthermore, a novel mapping algorithm (EnSite OT-near-field best duplicate) helps distinguish near-field potentials from far-field potentials by annotating the highest frequency component of the omnipolar electrograms.^2^ Recent reports have revealed that the high-peak-frequency (PF) potentials could reflect the critical isthmus site of the macroreentrant AT.^3^^,^^4^ However, little is known about the utility for ATP-sensitive AT with a microreentrant circuit. This conceptual study aimed to examine the possibility of identifying critical isthmus distribution for ATP-sensitive ATs, using the PF analysis.

## Methods

We initially identified 16 ATP-sensitive ATs, treated with catheter ablation at the National Cerebral and Cardiovascular Center, Suita, Japan, from September 2023 to February 2026. ATP sensitivity was confirmed by AT termination with prolongation of the atria-atria interval despite no changes in atria-His interval by the rapid intravenous injection ranging from 2.5 to 5.0 mg.^5^ The exclusion criteria were (1) the use of 3D mapping systems other than EnSite X (n = 4) and (2) unanalyzable cases with no identification of orthodromic capture owing to AT terminations (n = 5). 7 patients were enrolled based on the criteria and evaluated. Clinical characteristics are presented in Table 1. Electroanatomic maps were obtained using an Advisor HD Grid under the OT-near-field duplicate algorithm. We gradually increased the cutoff value of the emphasis map until a small dense area with higher PF values than those of the EAAS, defined as a “high-frequency cluster,” was distinguished.^6^ Distributions of high-frequency cluster and critical isthmus estimated through the responses of entrainment pacing and ablation were examined.

**Table 1:** Clinical characteristics of the study cohort patients

Case number	Age (y)	Sex	TCL (ms)	EAAS		Orthodromic capture pacing site	Successful ablation site	Number of RF applications	High-frequency cluster	
				Location	PF (Hz)				Location	PF (Hz)
1	70	Female	530	Posterior to the His bundle	426	Anterolateral right atrium	Lateral to EAAS	13	Lateral to EAAS	614 ± 37
2	56	Female	400	Septal to the His bundle	562	Coronary sinus ostium	Septal to EAAS	11	Septal to EAAS	636 ± 27
3	72	Female	460	Posterior to the His bundle	426	Noncoronary cusp	Noncoronary cusp	4	–	–
4	70	Female	410	Lateral to the His bundle	543	Anterolateral right atrium	Lateral to EAAS	8	Lateral to EAAS	575 ± 60
5	77	Male	630	Lateral to the His bundle	543	Anterolateral right atrium	Lateral to EAAS	11	Lateral to EAAS	666 ± 40
6	69	Female	380	Lateral to the His bundle	543	High lateral right atrium	Lateral to EAAS	6	Lateral to EAAS	657 ± 69
7	75	Female	490	Anterior mitral annulus (left atrium)	≥716	High anterior left atrium	Superior to EAAS	18	–	–

Clinical characteristics of the patients with adenosine triphosphate–sensitive atrial tachycardia were summarized. The orthodromic capture pacing site indicates the location where the pacing orthodromically captured the EAAS.

EAAS = earliest atrial activation site; PF = peak frequency; RF = radiofrequency; TCL = tachycardia cycle length.

This study was approved by the institutional research board of the National Cerebral and Cardiovascular Center (M26-148) and conducted with adherence to the Declaration of Helsinki. All patients provided a written informed consent before undergoing ablation.

## Results

### Discovery cohort

**Figure 1:**
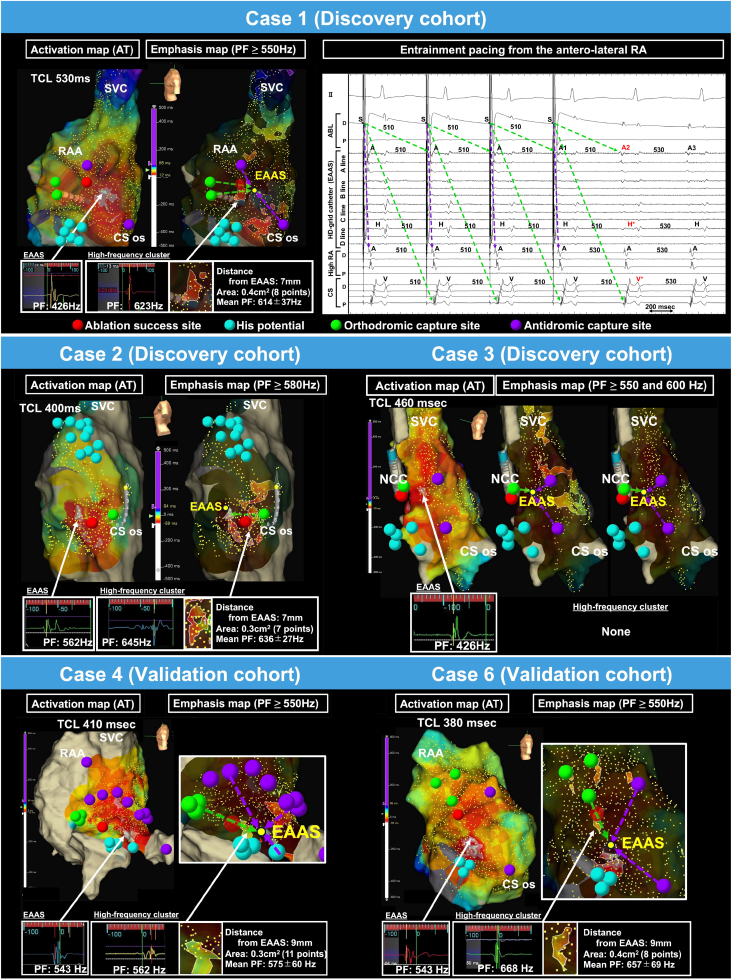
Activation and emphasis maps of adenosine triphosphate–sensitive atrial tachycardias. RA emphasis maps identified high-frequency clusters with higher PF potentials than the EAAS, except in case 3. Orthodromic capture of the EAAS was confirmed by pacing delivered from the direction in cases 1 and 2. A = atrial potential; ABL = ablation catheter; CS os = coronary sinus ostium; D = distal electrogram; EAAS = earliest atrial activation site; H = His potential; NCC = noncoronary cusp; P = proximal electrogram; PF = peak frequency; RA =right atrial; RAA = right atrial appendage; S = stimuli; SVC = superior vena cava; TCL = tachycardia cycle length; V = ventricular potential.

Cases 1 and 2 involved 70- and 56-year-old women with ATP-sensitive ATs. Obtained RA activation maps displayed centrifugal patterns with the EAAS (PF 426 Hz and 562 Hz) located adjacent to the His bundle potential recording sites. The emphasis map (cutoff 550 Hz and 580 Hz) retrospectively distinguished high-frequency clusters (case 1, 0.4 cm^2^, 8 points, 614 ± 37 Hz; case 2 0.3 cm^2^, 7 points, 636 ± 27 Hz) (Figure 1) on the line connecting the EAAS and the pacing sites, where orthodromic capture of the EAAS with an A2-H∗-V∗ response^7^ was obtained. The distance between the EAAS and the high-frequency cluster was 7 mm in both cases. The ATs were terminated by RF application at the high-frequency cluster sites.

In case 3 (72-year-old woman), orthodromic capture of the EAAS was confirmed by pacing from the noncoronary cusp only, where RF applications terminated the AT. The high-frequency cluster adjacent to the EAAS (PF 426 Hz) was not identified in the RA (Figure 1), despite using an emphasis map (cutoff value 450–600 Hz).

### Validation cohort

**Figure 2:**
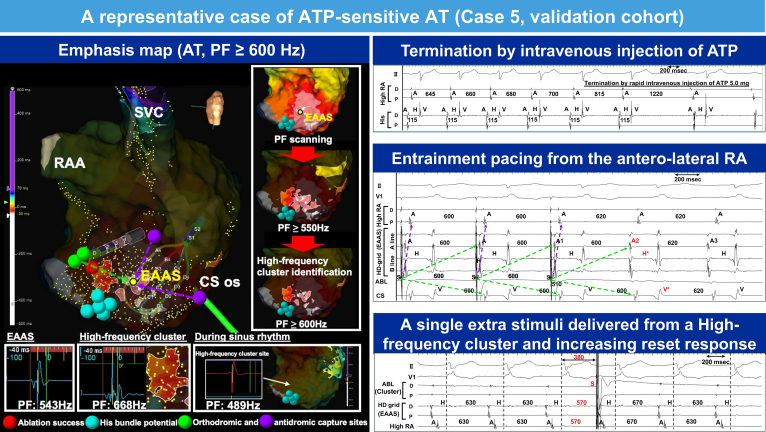
PF-guided electrophysiological study in a representative case. ATP sensitivity was confirmed by termination after the intravenous injection of 5.0 mg. An emphasis map highlighted a high-frequency cluster adjacent to the EAAS during AT. The orthodromic capture pacing site could be predicted from the distribution. The AT was reset and ablated at a high-frequency cluster site. A = atrial potential; ABL = ablation catheter; AT = atrial tachycardia; ATP = adenosine triphosphate; CS os = coronary sinus ostium; D = distal electrogram; EAAS = earliest atrial activation site; H = His potential; P = proximal electrogram; PF = peak frequency; RAA = right atrial appendage; S = stimuli; SVC = superior vena cava; TCL = tachycardia cycle length; V = ventricular potential.

In cases 4–6 (72 ± 4 years old; 2 women), we verified the utility of PF-guided electrophysiological study. Based on findings from the discovery cohort, we hypothesized that the orthodromic capture pacing site could be predicted from the distribution of high-frequency clusters. The emphasis maps (cutoff 550–600 Hz) intraoperatively identified high-frequency clusters (case 4, 0.3 cm^2^, 11 points, 575 ± 60 Hz; case 5, 0.5 cm^2^, 20 points, 666 ± 40 Hz; and case 6, 0.4 cm^2^, 8 points, 657 ± 69 Hz) (Figures 1 and 2) at the anterolateral area adjacent to the EAAS (PF 543 Hz). We attempted entrainment pacing from the direction and confirmed orthodromic capture of the EAAS potential. The ATs were reset by atrial single extra stimuli (output 5.0 V; pulse width 2.0 ms) and successfully ablated at high-frequency cluster sites. During sinus rhythm, no potentials with PF exceeding 550 Hz were observed at high-frequency cluster sites.

**Figure 3:**
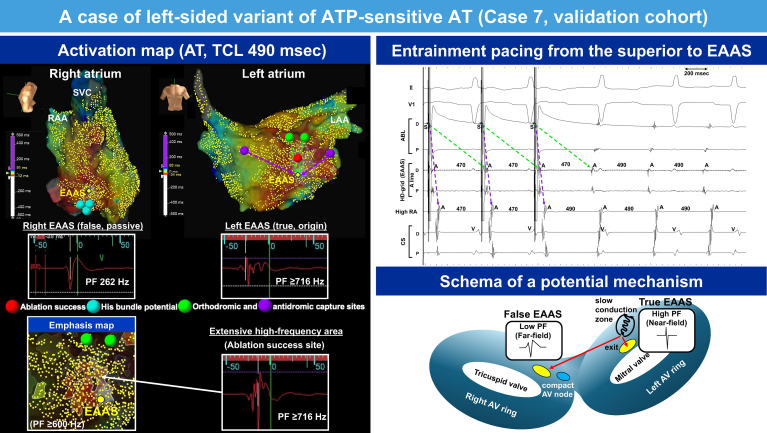
PF analysis in a case of left-sided variant of ATP-sensitive atrial tachycardia. The PF at the EAAS during ATP-sensitive AT was relatively low in the RA. An additional left atrial mapping revealed that the potentials preceded those of the RA. An emphasis map (PF ≥600 Hz) demonstrated an extremely high-frequency area extending superiorly from the true EAAS. Entrainment pacing and radiofrequency ablation were performed from the superior direction, confirming orthodromic capture of the EAAS and termination of the AT. A relatively low PF at the EAAS in the RA may indicate passive activation and prompt additional left atrial mapping to evaluate a left-sided variant of ATP-sensitive AT. A = atrial potential; ABL = ablation catheter; AT = atrial tachycardia; ATP = adenosine triphosphate; AV = atrioventricular; CS os = coronary sinus ostium; D = distal electrogram; EAAS = earliest atrial activation site; H = His potential; LAA = left atrial appendage; P = proximal electrogram; PF = peak frequency; RA = right atrium; RAA = right atrial appendage; S = stimuli; SVC = superior vena cava; TCL = tachycardia cycle length; V = ventricular potential.

In case 7 (75-year-old woman), the PF at the EAAS located adjacent to the His bundle potential recording site was low (262 Hz). Additional left atrial mapping revealed earlier activation, indicating a left-sided variant of ATP-sensitive AT.^8^ The true EAAS is distributed at the anterior mitral annulus, showing an extremely high PF (≥716 Hz, exceeding the analysis limit). An emphasis map (cutoff 600 Hz) demonstrated a high-frequency area extending superiorly from the EAAS. Therefore, entrainment pacing and RF applications were performed from the superior direction, confirming orthodromic capture of the EAAS and termination of the AT (Figure 3).

## Discussion

### Major findings

To the best of our knowledge, this is the first study to demonstrate the possible utility of PF-guided ablation for ATP-sensitive AT. We evaluated electroanatomic maps of ATP-sensitive ATs using the OT-near-field duplicate algorithm. The major findings were as follows: (1) among 6 typical ATP-sensitive ATs, a high-frequency cluster adjacent to the EAAS was identified in 5 cases, and (2) orthodromic capture of the EAAS potential could be confirmed by pacing from the direction of a high-frequency cluster, where AT was successfully ablated.

### Possibility of critical isthmus visualization

At the slow-conduction zone, fractionated electrograms with high- and low-PF components can be generated, given that the activation timings between near- and far-fields differ.^2^ Among cases of macroreentrant ATs, high-PF and low-voltage potentials reflect the critical isthmus site.^3^^,^^4^ Given that the atrioventricular ring tissue area involves calcium channel–dependent tissues^9^ and a small amount of myocardium,^10^ we hypothesized that the PF analysis can be applied for ATP-sensitive AT.

Our findings reveal the potential feasibility and utility of critical isthmus identification of ATP-sensitive AT. In our cohort, 83% of typical ATP-sensitive ATs had a high-frequency cluster. The high-frequency clusters were distributed on the line connecting the EAAS and orthodromic capture pacing site, with a mean proximity to the EAAS of 8 ± 1 mm. The ATs were reset and ablated at high-frequency cluster sites. Although further investigation is required, the absence of a high-frequency cluster or a relatively low PF at the EAAS in the RA may warrant additional mapping of the left atrium or the noncoronary cusp.

### Clinical implication

RF applications from the direction of identified high-frequency cluster potentially provide an effective solution and sufficient distance from the cardiac conduction system in cases where entrainment pacing repeatedly terminates ATP-sensitive AT.

### Limitations

This study has a limited number of ATP-sensitive AT cases, evaluated using a 3D mapping system capable of assessing the PF values. PF values depend on several factors, including the amount of myocardium, the distance between the recorded potentials and electrodes, catheter contact, and local conduction velocity. Further studies should validate the feasibility and utility of PF-guided catheter ablation strategies for ATP-sensitive AT.

## Conclusion

This conceptual study demonstrates the potential utility of critical isthmus identification for ATP-sensitive AT as a high-frequency cluster, using the OT-near-field duplicate algorithm.

## Disclosures

Kengo Kusano received speaker honoraria from Daiichi Sankyo Company and Medtronic and research grants from Medtronic and JSR outside the submitted work. Koji Miyamoto received funding, grants, and speaker honoraria from Medtronic, Biosense Webster, Abbott, and Boston Scientific outside the submitted work. He is also affiliated with a department endowed by Medtronic outside the submitted work. Tsukasa Kamakura received speaker honoraria from Biosense Webster, Medtronic, and Boston Scientific outside the submitted work. Nobuhiko Ueda and Satoshi Oka received speaker honoraria from Medtronic outside the submitted work. Akinori Wakamiya received speaker honoraria from Biosense Webster, Medtronic, and BIOTRONIK outside the submitted work. The other authors had no conflicts of interest to declare.
