# Supplementary material for: Peak frequency analysis to identify critical isthmus distribution for adenosine triphosphate–sensitive atrial tachycardia
Source: Heart Rhythm O2. 2026 Apr 15;7(7):1387–91. doi: 10.1016/j.hroo.2026.04.006 (PMC13389971; doi:10.1016/j.hroo.2026.04.006)
Supplement: Supplementary Legends [file mmc2.docx]

Video legend

A video demonstration of stepwise peak frequency-guided catheter ablation for adenosine triphosphate-sensitive atrial tachycardias.
